# Long non-coding RNA (lncRNA) transcriptional landscape in breast cancer identifies LINC01614 as non-favorable prognostic biomarker regulated by TGFβ and focal adhesion kinase (FAK) signaling

**DOI:** 10.1038/s41420-019-0190-6

**Published:** 2019-06-24

**Authors:** Radhakrishnan Vishnubalaji, Hibah Shaath, Eyad Elkord, Nehad M. Alajez

**Affiliations:** Cancer Research Center, Qatar Biomedical Research Institute (QBRI), Hamad Bin Khalifa University (HBKU), Qatar Foundation (QF), PO Box 34110 Doha, Qatar

## Abstract

Long non-coding RNAs (lncRNAs) represent a class of epigenetic regulators implicated in a number of physiological and pathological conditions. Herein, we characterized the lncRNA expression portrait from 837 patients with invasive breast cancer and 105 normals from the cancer genome atlas (TCGA), which revealed eighteen upregulated and forty-six downregulated lncRNAs. Clustering analysis revealed distinct lncRNA profile for the triple negative breast cancer (TNBC) and normal breast tissue, while less separation was observed among the HER2^+^HR^+^, HER2^+^HR^−^, HER2^−^HR^+^ molecular subtypes. LINC01614, and LINC01235 correlated with worse disease-free survival (DFS), while the expression of lnc-LRR1–1, lnc-ODF3B-2, AC015712.5, lnc-LAMB3–1, lnc-SPP2–3, and lnc-MAP9–2 correlated with better DFS. The expression of LINC01235 correlated with worse overall survival (OS), while the expression of MIR205HG, lnc-MAP2K6–5, FGF14-AS2, lnc-SPP2–3 correlated with better OS. Highest expression of LINC01614 was observed in progesterone receptor (PR)^+^, Estrogen receptor (PR)^+^, and HER2^+^ tumors, while lowest expression was in TNBC. Concordantly, LINC01614 was highly expressed in the luminalB/HER2^+^ subtype from the SRP062132 dataset. Elevated expression of LINC01614 was subsequently validated in primary breast cancer tissue and breast cancer cell lines. Bioinformatics and pathway analyses on LINC01614^high^ vs. LINC01614^low^ BC tissue revealed TGFβ1 and ECM as the most activated networks in LINC01614^high^ tumors. Concordantly, strong correlation between the expression of LINC01614 and COL10A1 (*R*^2^ = 0.6929), SPOCK1 (*R*^2^ = 0.5156), ZEB1 (*R*^2^ = 0.3372), TGFBI (*R*^2^ = 0.2978), TGFB1 (*R*^2^ = 0.1985), ACTA2 (*R*^2^ = 0.1833), and TAGLN (*R*^2^ = 0.1909) was observed. Mechanistically, exogenous TGFB1 induced LINC01614 expression in the BT474 triple positive BC model, while small-molecule inhibition of transforming growth factor β (TGFβ, SB-431542) or focal adhesion kinase (FAK, PF-573228) abrogated LINC01614 expression. Our data revealed the lncRNA transcription landscape in breast cancer and its molecular subtypes. Our data provide novel insight implicating LINC01614 as unfavorable prognostic marker in BC, its association with the HR^+^/HER2^+^ BC molecular subtype and its regulation by TGFβ and FAK signaling.

## Introduction

Breast cancer (BC) is the most common cancer type in females worldwide^1^. The molecular mechanisms involved in BC pathogenesis have been thoroughly studied, leading to BC classification into three major subtypes: Luminal which is positive for estrogen (ER+) and progesterone receptors (PR+), human epidermal growth factor receptor 2 (HER2+), and basal-like tumors, which lacks hormone receptor and HER2 expression, called triple-negative breast cancer (TNBC)^2–4^. While this classification has led to better stratification of BC, this disease is still associated with high mortality rate underscoring a need to develop novel molecular signature for better stratification and for prediction of disease outcome.

Although a number of gene-expression signatures have been reported as diagnostic and prognostic biomarkers in BC, the role of non-coding RNAs in this domain is just beginning to unfold. Genome wide transcriptome studies have revealed the existence of large number of long non-coding RNAs (≥200 nucleotides)^5,6^. Current GENCODE database (version 30) revealed the existence of approximately 16,193 lncRNAs and 14,706 pseudogenes in the human genome.

LncRNAs are involved in regulating various biological processes, including tumor-suppressor and oncogenic pathways and may serve as prognostic markers in BC. A number of oncogenic (H19, SRA, LSINCT5, Zfas1, lncRNA-Smad7, LOC554202, HOTAIR, SOX2OT and FAL1) and tumor suppressor (GAS5 and XIST) lncRNAs have been identified in BC; however their regulation and the mechanisms of action for the majority of lncRNAs remains to be unraveled^6,7^.

In this study, we characterized the lncRNA expression portrait from 837 patients with invasive BC and 105 normal breast tissues from the cancer genome atlas (TCGA) breast cancer dataset.

Our comparative analysis identified eighteen upregulated and forty-six downregulated lncRNAs in breast cancer compared to normal tissue. Interestingly, we identified eleven lncRNAs: LINC01614, LINC01235, lnc-LRR1–1, lnc-ODF3B-2, AC015712.5, lnc-LAMB3–1, lnc-SPP2–3, lnc-MAP9–2, MIR205HG, lnc-MAP2K6–5 and FGF14-AS2 to be associated with BC patient outcome. In particular, our data provided novel insight implicating LINC01614 as unfavorable prognostic marker in BC, its association with the HR^+^/HER2^+^ BC molecular subtype and its regulation by TGFβ and FAK signaling.

## Materials and methods

### Data analyses and bioinformatics

Long noncoding RNA (lncRNA) expression from 837 invasive breast carcinoma and 105 normal subjects were retrieved from The Atlas of Noncoding RNAs in Cancer (TANRIC; http://bioinformatics.mdanderson.org/main/TANRIC:Overview) database. Expression data were subsequently imported into Altanalyze v.2.1.0 software as described before^8^. Hierarchical clustering was performed using cosine for columns and cosine for rows while principal component analysis was performed to assess the relatedness of samples. Gene expression for the same cohort was retrieved from the cBioPortal for Cancer Genomics (https://www.cbioportal.org/) database as we described before^9^.

### RNA-Seq data analysis

Raw RNA sequencing data were retrieved from sequence read archive (SRA) database under accession no. SRP062132. Data were retrieved using the SRA toolkit version 2.9.2 as previously described^10^. Pair end reads were aligned to the hg19 human reference genome in CLC Genomics Workbench-12 (QIAGEN, Germany). The abundance of the expression of transcripts was measured as the score of TPM (Transcript Per Kilobase Million) mapped reads in CLC Genomics Workbench 12. Expression of LINC01614 in each molecular subtype was plotted using Graphpad Prism 6.0 software (Graphpad ® Software, San Diego, CA, USA)

### Gene set enrichment and modeling of gene interactions networks

Upregulated genes in the LINC01614^high^ BC group were imported into the Ingenuity Pathways Analysis (IPA) software (Ingenuity Systems; www.ingenuity.com/) and were subjected to functional annotations and regulatory network analysis using upstream regulator analysis (URA), downstream effects analysis (DEA), mechanistic networks (MN) and causal network analysis (CNA) prediction algorithms. IPA uses precise algorithm to predict functional regulatory networks from gene expression data and provides a significance score for each network according to the fit of the network to the set of focus genes in the database. The p value is the negative log of P and represents the possibility that focus genes in the network being found together by chance^11^.

### Statistical and survival analysis

Kaplan-Meir survival analysis and plotting were conducted using IBM SPSS statistics version 24 software. For survival analysis, patients were grouped into high or low based on LINC01614 log2 gene expression. The log-rank test was used to compare the outcome between expression groups. Statistical analyses to compare specific gene expression and graphing were performed using Graphpad Prism 6.0 software. Unpaired two-tailed *t*-test and *p* value of <0.05 was considered significant as we described before^12^.

### Cell culture, recombinant TGFβ treatment, and small molecule inhibition

Human breast cancer cell lines (BT474, T47D, MDAMB453, ZR751, MCF7, HCC70, HS578T, MDAMB468, BT549 and MDAMB231) were cultured in Dulbecco’s modified Eagle’s medium/RPMI 1640 supplemented with D-glucose 4500 mg/l, 4 mM L-glutamine and 110 mg/l sodium pyruvate, 10% fetal bovine serum and 1x penicillin–streptomycin (Pen-Strep) (all purchased from Gibco-Invitrogen, Waltham, MA, USA). The triple positive BC cell line (BT474) was treated with rhTGFβ (10 ng/ml, Peprotec, London, UK), TGFβ inhibitor (SB-431542; 10 μM, Selleckchem Inc., Houston, TX, USA), FAK inhibitor (PF-573228; 5 μM, Selleckchem Inc., Houston, TX, USA) and combination of rhTGFβ and TGFβ inhibitor. Pharmacological inhibition of TGF-β and FAK pathways were conducted as we previously described^9,13^. Briefly, 0.2 × 10^6^ cells/well were cultured in 6 well plates (duplicate) and incubated for 48 hours and subsequently the expression of LINC01614 was measured using qRT-PCR. Assays were carried out with appropriate DMSO control.

### LncRNA validation using qRT-PCR

Tumor tissue (TT) specimens from eight BC tissue and adjacent normal tissue (NT) were obtained from treatment-naive BC patients prior to surgery with a proper written informed consent. The study was approved by Qatar Biomedical Research Institute, Doha, Qatar (Protocol no. 2017–006). Total RNA was extracted from eight primary BC tissue, adjacent normal tissue, and from a panel of breast cancer cell lines using Norgon RNA/DNA/Protein Purification Plus Kit (Norgen Biotek Corp, Ontario, Canada) as per the manufacturer’s instructions. Expression level of LINC01614 was validated using SYBR Green-based quantitative reverse transcriptase-polymerase chain reaction (qRT-PCR). The total RNA (500 ng) was reverse transcribed into complementary DNA (cDNA) using a High Capacity cDNA Reverse Transcript Kit (catalogue No. 4368814; ABI) according to the manufacturer’s protocol. Relative levels of lncRNA was determined using the cDNA as template in real-time PCR analysis using the Applied Biosystems QuantStudio 6/7 Flex Real-time PCR system. Primer sequences used in the current study were: LINC01614 F: 5′-AACCAAGAGCGAAGCCAAGA-3′; LINC01614 R: 5′-GCTTGGACACAGACCCTAGC-3′; GAPDH F: 5′-CTGGTAAAGTGGATATTGTTGCCAT-3′; and GAPDH R: 5′-TGGAATCATATTGGAACATGTAAACC-3′. The relative expression level was calculated using –ΔΔCT, GAPDH was used as an endogenous control.

## Results

### Expression profiling of lncRNAs from the TCGA BC dataset compared to normal breast tissue

**Fig. 1:**
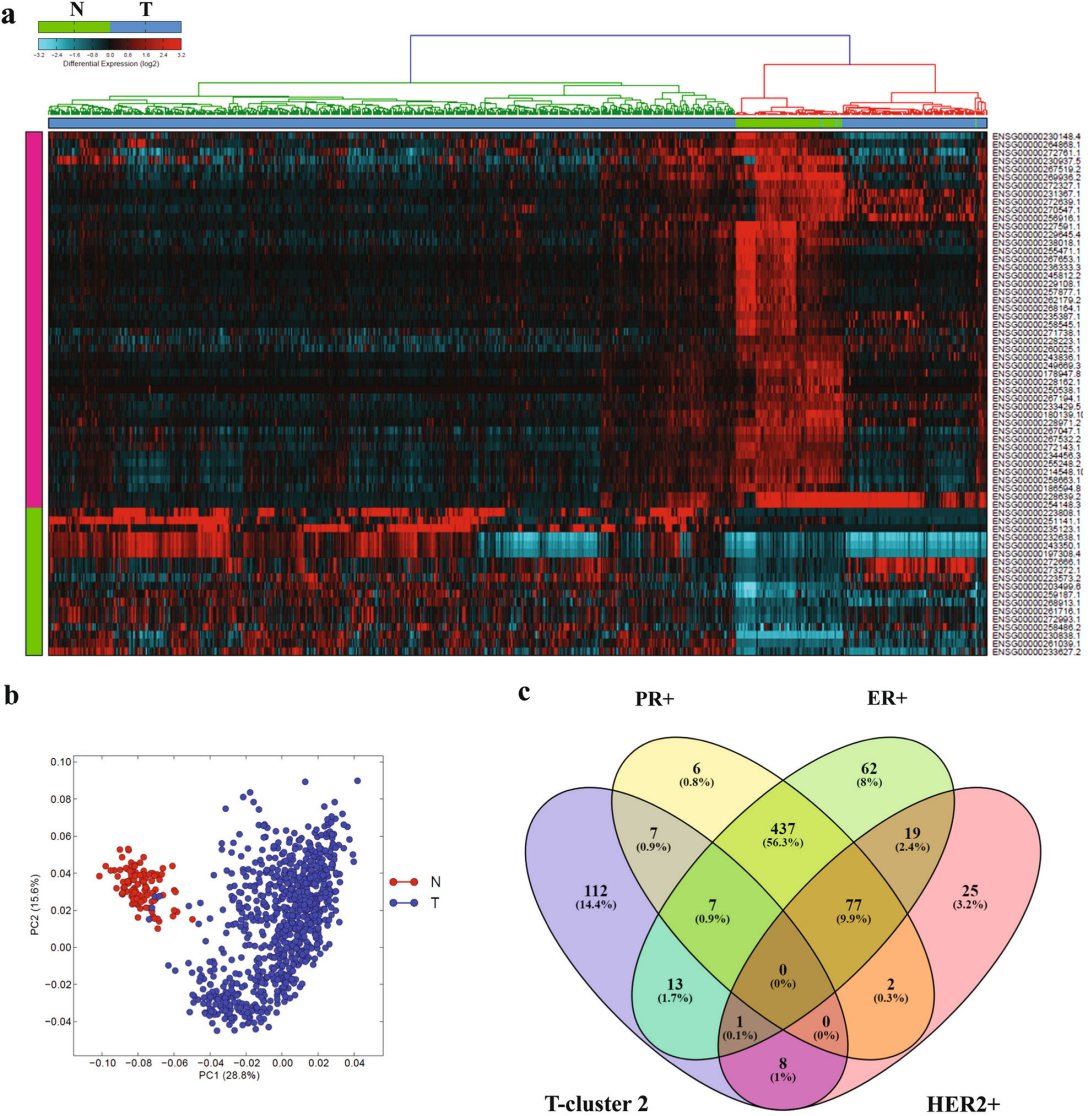
lncRNA transcriptional portrait in breast cancer compared to normal tissue. **a** Hierarchical clustering of breast cancer (*n* = 837) and normal (*n* = 105) breast tissue based on differentially expressed lncRNA transcripts. Each column represents one sample and each row represents a transcript. Expression level of each transcript (log2) in a single sample is depicted according to the color scale. **b** Principal component analysis (PCA) for the lncRNA transcriptome of breast cancer and normal breast tissue. **c** Venn diagram depicting the overlap between tumors samples from tumor cluster 2 (T-cluster 2) on the further right of the heatmap in panel (a) and the indicated breast cancer molecular subtype

Expression data for 12727 lncRNAs from 837 patients with invasive BC and 105 normal breast tissue were retrieved from TANRIC database and were subjected to differential expression analysis, which identified 18 upregulated and 46 downregulated lncRNAs (≤2≥, FDR *p* ≤ 0.05; Table 1). Hierarchical clustering revealed three major clusters, where breast cancer samples clustered at both sides while normals clustered in the middle (Fig. 1a). Principle component analysis (PCA) also revealed clear separation of normal from breast cancer based on lncRNA expression (Fig. 1b). Interestingly, we observed majority of patients in the further right cluster (76%; Fig. 1a) to be of the TNBC molecular subtype (Fig. 1c).

**Table 1:** Differentially expressed lncRNAs in breast cancer vs normal

Ensembl Gene ID	LNCipedia gene ID	log fold Tum vs Nor	fold Tum vs Nor	Tum vs Nor p value (raw)	Tum vs Nor p value (adj)	Status
ENSG00000230838.1	LINC01614	2.341821475	5.069422738	5.65E-81	3.76E-79	Up
ENSG00000203499.6	lnc-MAPK15–6	1.767788642	3.405315908	3.82E-82	2.61E-80	Up
ENSG00000223808.1	AC044784.1, lnc-GATA3–7	1.610083258	3.052694584	1.25E-16	1.13E-15	Up
ENSG00000259187.1	lnc-TRIM69–1	1.590879481	3.01232928	1.81E-44	4.73E-43	Up
ENSG00000261039.1	LINC02544	1.398432474	2.636150018	7.47E-43	1.85E-41	Up
ENSG00000258486.2	lnc-LRR1–1	1.341780081	2.534638644	9.58E-26	1.33E-24	Up
ENSG00000235123.1	DSCAM-AS1	1.326866563	2.508572377	6.20E-13	4.46E-12	Up
ENSG00000232638.1	lnc-TAF3–1	1.21241703	2.317255348	1.40E-12	9.85E-12	Up
ENSG00000272993.1	lnc-HIST2H2AA4–1	1.185802408	2.274898863	1.78E-37	3.74E-36	Up
ENSG00000243350.1	GATA3-AS1	1.148653771	2.217069154	3.27E-13	2.39E-12	Up
ENSG00000233627.2	C4A-AS1	1.105244735	2.1513537	7.23E-16	6.20E-15	Up
ENSG00000272666.1	lnc-ODF3B-2	1.077677457	2.110635509	1.89E-19	1.98E-18	Up
ENSG00000273272.1	lnc-KLHDC7B-2	1.048411607	2.06825147	4.74E-19	4.87E-18	Up
ENSG00000197308.4	GATA3-AS1	1.044585641	2.062773821	9.84E-14	7.48E-13	Up
ENSG00000223573.2	lnc-TINCR-1	1.044336743	2.062417976	1.83E-17	1.73E-16	Up
ENSG00000261716.1	lnc-HIST2H2AA3–1	1.026047258	2.036437106	3.92E-35	7.61E-34	Up
ENSG00000251141.1	MRPS30-DT	1.02054162	2.028680429	1.58E-07	7.36E-07	Up
ENSG00000268913.1	lnc-KCNK6–1	1.001295226	2.001796371	8.85E-23	1.07E-21	Up
ENSG00000228223.1	HCG11	−1.017371351	−2.024227373	2.51E-53	8.53E-52	Down
ENSG00000235387.1	na	−1.020539284	−2.028677144	1.96E-59	7.71E-58	Down
ENSG00000228162.1	lnc-SPP2–3	−1.043000128	−2.060508089	9.21E-115	1.41E-112	Down
ENSG00000272639.1	AC015712.5	−1.05048961	−2.071232647	1.95E-49	5.91E-48	Down
ENSG00000256916.1	lnc-BIRC2–4	−1.069704648	−2.09900361	2.70E-26	3.84E-25	Down
ENSG00000264868.1	lnc-STEAP4–1	−1.080167322	−2.114281279	8.30E-28	1.25E-26	Down
ENSG00000250538.1	lnc-MAP9–2	−1.096920898	−2.138976893	1.05E-104	1.21E-102	Down
ENSG00000243836.1	WDR86-AS1	−1.151180613	−2.220955695	1.05E-116	1.73E-114	Down
ENSG00000178947.8	SMIM10L2A	−1.161204888	−2.236441294	2.98E-104	3.39E-102	Down
ENSG00000267194.1	lnc-MAP2K6–5	−1.162956703	−2.239158576	5.17E-82	3.52E-80	Down
ENSG00000233429.5	HOTAIRM1	−1.179118648	−2.264384021	1.06E-87	8.10E-86	Down
ENSG00000271738.1	lnc-TSPAN14–1	−1.191983318	−2.284666075	1.60E-57	6.01E-56	Down
ENSG00000214548.10	lnc-DLK1–35	−1.199354187	−2.296368526	4.55E-110	6.30E-108	Down
ENSG00000268164.1	na	−1.204434904	−2.304469867	2.30E-93	2.01E-91	Down
ENSG00000260025.1	lnc-FEZ2–7	−1.20586992	−2.306763212	5.20E-63	2.24E-61	Down
ENSG00000245812.2	LINC02202	−1.227351365	−2.341367447	7.20E-190	9.16E-187	Down
ENSG00000262179.2	MYMX	−1.233789324	−2.351839037	8.67E-99	8.49E-97	Down
ENSG00000186594.8	MIR22HG	−1.237175737	−2.357365953	4.69E-86	3.43E-84	Down
ENSG00000270547.1	LINC01235	−1.239824501	−2.361698012	6.31E-42	1.50E-40	Down
ENSG00000255248.2	MIR100HG	−1.261015808	−2.396644303	3.69E-100	3.82E-98	Down
ENSG00000272143.1	FGF14-AS2	−1.285565793	−2.437776382	8.61E-100	8.62E-98	Down
ENSG00000236333.3	TRHDE-AS1	−1.286805825	−2.439872611	8.25E-157	3.50E-154	Down
ENSG00000257877.1	lnc-MAPKAPK5–1	−1.291455467	−2.447748728	4.55E-139	1.16E-136	Down
ENSG00000267532.2	lnc-SLC16A11–7	−1.332087847	−2.517667645	7.47E-147	2.50E-144	Down
ENSG00000234456.3	MAGI2-AS3	−1.339265343	−2.530224405	1.04E-159	4.58E-157	Down
ENSG00000272327.1	lnc-NRG1–3	−1.352076922	−2.552793645	8.52E-68	4.13E-66	Down
ENSG00000258545.1	RHOXF1-AS1	−1.356631857	−2.560866164	6.26E-114	9.16E-112	Down
ENSG00000267653.1	lnc-ABCA5–6	−1.372853753	−2.589823452	1.55E-108	2.04E-106	Down
ENSG00000229108.1	LINC02587	−1.395635686	−2.631044566	6.69E-164	3.41E-161	Down
ENSG00000230148.4	HOXB-AS1	−1.426672474	−2.68825962	2.86E-42	6.89E-41	Down
ENSG00000228971.2	lnc-RWDD3–5	−1.450759321	−2.733518843	2.47E-108	3.21E-106	Down
ENSG00000249669.3	CARMN	−1.474915257	−2.779673159	1.36E-234	1.73E-230	Down
ENSG00000230937.5	MIR205HG	−1.500142384	−2.828706285	1.10E-27	1.65E-26	Down
ENSG00000180139.10	ACTA2-AS1	−1.563395501	−2.955486233	1.37E-118	2.33E-116	Down
ENSG00000231367.1	lnc-ATL2–1	−1.570416598	−2.969904619	4.73E-83	3.27E-81	Down
ENSG00000258663.1	lnc-RTL1–1	−1.573129653	−2.975494916	3.68E-96	3.40E-94	Down
ENSG00000255471.1	lnc-FZD4–1	−1.665847283	−3.172999476	1.39E-151	5.52E-149	Down
ENSG00000267519.2	lnc-C19orf57	−1.684869673	−3.215113511	2.96E-81	1.98E-79	Down
ENSG00000267047.1	lnc-SLC16A11–7	−1.783333068	−3.442205117	1.36E-109	1.85E-107	Down
ENSG00000238018.1	lnc-RTN4–3	−1.826091508	−3.545751691	7.48E-143	2.21E-140	Down
ENSG00000272761.1	lnc-CCDC80–5	−1.868752854	−3.652167299	2.80E-47	7.92E-46	Down
ENSG00000229645.4	lnc-SYNE3–1	−2.025990744	−4.072714669	5.48E-134	1.32E-131	Down
ENSG00000228639.2	lnc-SLC39A11–10	−2.048436659	−4.136574768	6.54E-44	1.68E-42	Down
ENSG00000254148.3	lnc-SLC39A11–10	−2.181617437	−4.536618794	6.46E-45	1.71E-43	Down
ENSG00000227591.1	lnc-LAMB3–1	−2.192798277	−4.571914035	1.67E-169	1.12E-166	Down
ENSG00000269936.2	CARMN	−2.778459057	−6.861191136	5.33E-170	3.77E-167	Down

### Identification of lncRNAs associated with overall survival (OS) and disease-free survival (DFS) in the TCGA BC dataset

**Fig. 2:**
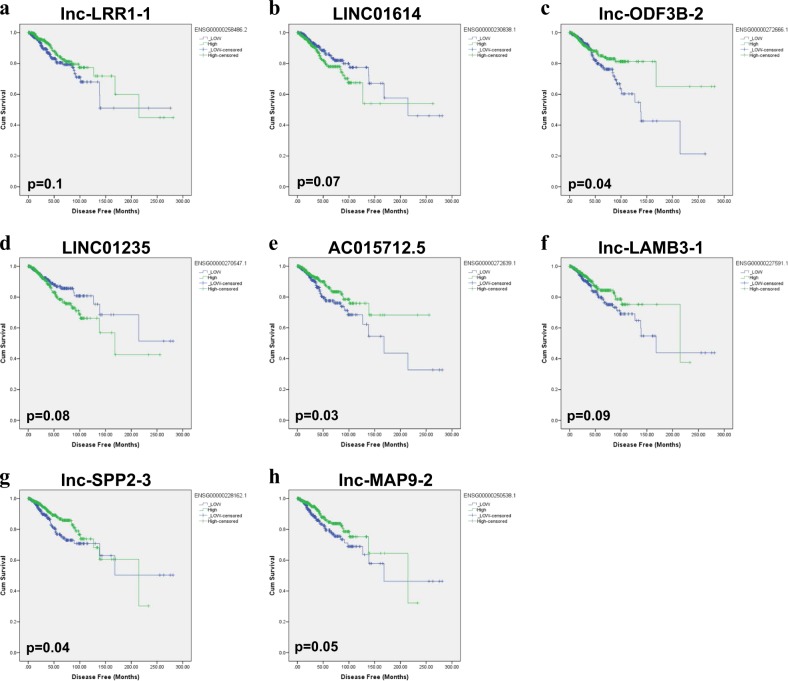
Disease-free survival (DFS) of breast cancer patients based on lncRNA expression. Kaplan-Meir DFS analysis for lnc-LRR1–1 (**a**), LINC01614 (**b**), lnc-ODF3B-2 (**c**), LINC01235 (**d**), AC015712.5 (**e**), lnc-LAMB3–1 (**f**), lnc-SPP2–3 (**g**), and lnc-MAP9–2 (**h**) in the TCGA BC cohort. Significance was calculated using the log-rank test. *p* values are indicated on each plot

**Fig. 3:**
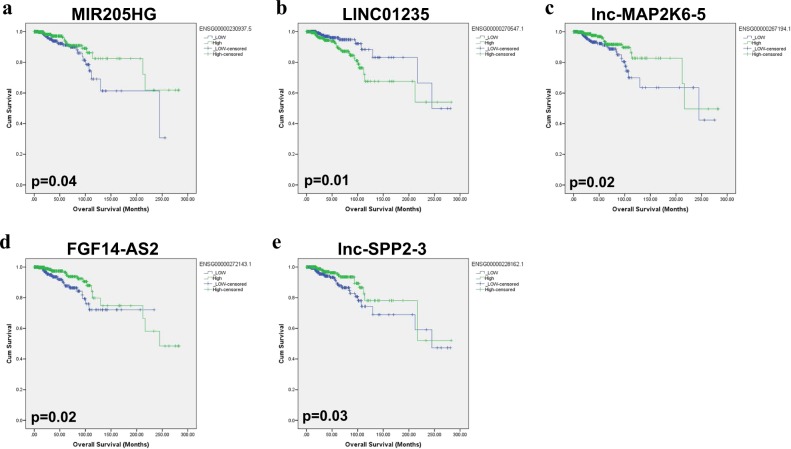
Overall survival (OS) of breast cancer patients based on lncRNA expression. Kaplan-Meir OS analysis for MIR205HG (**a**), LINC01235 (**b**), lnc-MAP2K6–5 (**c**), FGF14-AS2 (**d**), and lnc-SPP2–3 (**e**) in the TCGA BC cohort. Significance was calculated using the log-rank test. *p* values are indicated on each plot

The differentially expressed lncRNAs from the profiling study were subsequently subjected to Kaplan-Meir survival analysis. Our data revealed LINC01614 and LINC01235 to predict worse DFS of BC patients (Fig. 2b and d; log-rank test = 0.07 and 0.08, respectively). On the other hand, lnc-LRR1–1, lnc-ODF3B-2, AC015712.5, lnc-LAMB3–1, lnc-SPP2–3, and lnc-MAP9–2 were associated with better DFS (Fig. 2a, c, e–h). Interestingly, the expression of LINC01235 correlated with worse DFS and worse OS (Figs. 2d and 3b), while the expression of MIR205HG, lnc-MAP2K6–5, FGF14-AS2, lnc-SPP2–3 correlated with better OS (Fig. 3a, c–e). Taken together, our data revealed LINC01614 as the only upregulated lncRNAs in BC and associated with worse DFS in the TCGA dataset.

### LINC01614 expression correlates with HER2^+^HR^+^ invasive breast cancer molecular subtype

**Fig. 4:**
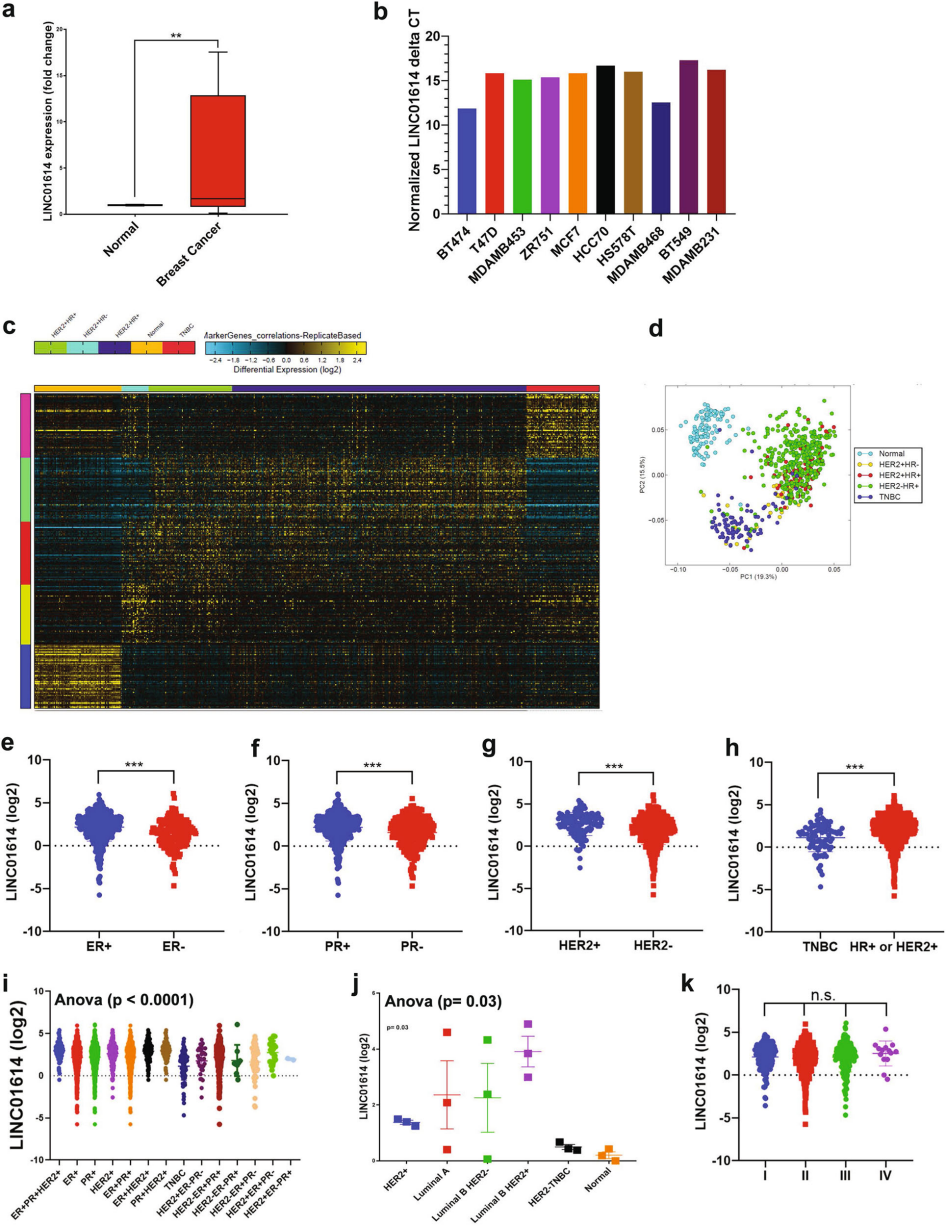
Correlation between the expression of LINC01614 and BC molecule subtype. **a** Expression of LINC01614 in eight BC patients (red box) and adjacent normal tissue (blue box) using qRT-PCR. Data are presented as mean ± S.E., *n* = 8 for each group. **b** Expression of LINC01614 in a panel of BC cell lines. Data are presented as normalized delta CT expression value. **(c)** Heat map clustering of BC (*n* = 837) and normal (*n* = 105) breast tissue based on molecular subtypes of BC (HER2 + HR+, HER2+HR−, HER2-HR+, TNBC, and normal). Each row represents expression level of the indicated lncRNA (log2). Expression level of transcriptomes in a single sample is depicted according to the color scale (blue to yellow). (**d**) Principal component analysis (PCA) for the indicated BC molecular subtypes and normal breast tissue. Expression of LINC01614 in relation to estrogen receptor (ER, **e**), progesterone receptor (PR, **f**), and HER2 (**g**) status. **h** Expression of LINC01614 in TNBC vs non-TNBC BC patients. **i** Expression of LINC01614 in the indicated molecular subtype subgroups. **j** Expression of LINC01614 in the SRP062132 dataset in relation to the indicated molecular subtype. **k** Expression of LINC01614 in relation to the tumor stages

LINC01614 was the most highly expressed lncRNA (5.0 FC, *p* (adj) = 3.7 × 10^–79^) in breast cancer compared to normal tissue. We subsequently validated the expression of LINC01614 in a cohort of breast cancer patients, which revealed elevated expression of LINC01614 in BC compared to adjacent normal tissue (5.9 FC, *p* = 0.0007, Fig. 4a). Similarly, LINC01614 expression was detected in a panel of BC cell lines, where highest expression was observed in the BT474 triple positive BC cell line (Fig. 4b). We subsequently sought to determine if lncRNA expression can discriminate breast cancer with various molecular subtypes. To that end, the 837 BC samples were divided into HER2^+^HR^+^, HER2^+^HR^−^, HER2^−^HR^+^, and TNBC and were subjected to the marker finder algorithm in Altanalyze v.2.1.0 compared to 115 normal breast tissue. Clustering analysis revealed distinct molecular subtype for the TNBC and normal breast tissue, while less clear separation was observed among the three other molecular subtypes (HER2^+^HR^+^, HER2^+^HR^−^, HER2^−^HR^+^, Figs. 4c, d). The lncRNA profile distinctive for each molecular subtype is shown in Table 2. Interestingly, the expression of LINC01614 was highest in the HR^+^HER2^+^, while lowest expression was observed in TNBC molecular subtype (Fig. 4e–i). The expression of LINC01614 was subsequently validated in another cohort (SRP062132), which concordantly revealed highest expression in the luminal B/HER2^+^ molecular subtype (Fig. 4j). There was no difference in the expression of LINC01614 in relation to tumor stage (Fig. 4k).

**Table 2:** lncRNAs enriched according to breast cancer molecular subtype

TNBC		HER2-HR+		HER2+HR+		HER2+HR^−^		Normal	
**Ensembl gene ID**	**LNCipedia gene ID**	**Ensembl gene ID**	**LNCipedia gene ID**	**Ensembl Gene ID**	**LNCipedia gene ID**	**Ensembl gene ID**	**LNCipedia gene ID**	**Ensembl gene ID**	**LNCipedia gene ID**
ENSG00000263680.2	lnc-SOX9–4	ENSG00000248008.2	NRAV	ENSG00000266469.1	lnc-CDK12–2	ENSG00000232044.4	LINC01105	ENSG00000264016.2	lnc-TMEM220–1
ENSG00000227036.2	lnc-SLC39A11–1	ENSG00000249042.1	lnc-ANKRD34B-2	ENSG00000270145.1	lnc-CDC6–1	ENSG00000256940.1	lnc-FKBP2–1	ENSG00000234456.3	MAGI2-AS3
ENSG00000225177.1	lnc-CCDC28A-1	ENSG00000269619.1	lnc-C5AR2–3	ENSG00000266999.1	lnc-RDM1–1	ENSG00000261235.1	lnc-MPHOSPH6–1	ENSG00000241684.1	ADAMTS9-AS2
ENSG00000229124.2	VIM-AS1	ENSG00000267348.2	GEMIN7-AS1	ENSG00000272264.1	lnc-TPD52–1	ENSG00000251179.1	TMEM92-AS1	ENSG00000263400.2	
ENSG00000258910.2	LINC01956	ENSG00000263214.1	lnc-ZSCAN10–3	ENSG00000263874.1		ENSG00000249700.4	SRD5A3-AS1	ENSG00000267532.2	lnc-SLC16A11–7
ENSG00000246334.2	PRR7-AS1	ENSG00000248360.3	LINC00504	ENSG00000230838.1	LINC01614	ENSG00000257086.1	lnc-PPP1R14B-1	ENSG00000262097.1	LINC02185
ENSG00000272524.1	lnc-KLF11–3	ENSG00000235106.4	lnc-BRD3OS-1	ENSG00000263466.1	lnc-FBXO47–2	ENSG00000273257.1	lnc-SRD5A3–1	ENSG00000267047.1	lnc-SLC16A11–7
ENSG00000258938.1	lnc-RALGAPA1–1	ENSG00000263011.1	lnc-ZNF205–1	ENSG00000228265.1	RALY-AS1	ENSG00000235491.1	LINC01889	ENSG00000235904.1	RBMS3-AS3
ENSG00000224167.1	lnc-FAM19A3–2	ENSG00000225361.3	PPP1R26-AS1	ENSG00000261005.1	lnc-MLLT6–1	ENSG00000204603.2	LINC01257	ENSG00000255090.1	MIR100HG
ENSG00000227392.1	HPN-AS1	ENSG00000205913.2	SRRM2-AS1	ENSG00000228613.1	lnc-PXDN-3	ENSG00000234378.1	lnc-LAPTM4A-2	ENSG00000269936.2	CARMN
ENSG00000215866.3	LINC01356	ENSG00000231439.3	WASIR2	ENSG00000250917.1	lnc-AHCY-1	ENSG00000257588.1	lnc-RACGAP1–1	ENSG00000267069.1	lnc-CIDEA-1
ENSG00000254035.1	lnc-ZNF454–1	ENSG00000261801.1	LOXL1-AS1	ENSG00000260228.1	lnc-SLC38A8–2	ENSG00000231298.2	MANCR	ENSG00000254847.1	lnc-DKK3–2
ENSG00000237517.4	lnc-DGCR6–6	ENSG00000268913.1	lnc-KCNK6–1	ENSG00000203635.2	lnc-PXDN-1	ENSG00000236345.1	lnc-PHF3–1	ENSG00000215386.6	MIR99AHG
ENSG00000254615.2	lnc-OXR1–1	ENSG00000259459.1	LINC02568	ENSG00000253917.3	lnc-OTOP1–6	ENSG00000248359.1	lnc-CCDC125–6	ENSG00000226005.3	lnc-PFKP-16
ENSG00000272192.1	lnc-MTFR1–1	ENSG00000261664.1	TTC39A-AS1	ENSG00000263975.1	lnc-PCGF2–1	ENSG00000266222.1	lnc-HOXB1–1	ENSG00000249669.3	CARMN
ENSG00000229891.1	LINC01315	ENSG00000268204.1	lnc-PCP2–1	ENSG00000257702.3	lnc-TTC31–4	ENSG00000204832.5	ST8SIA6-AS1	ENSG00000244513.2	lnc-ARL6IP5–1
ENSG00000261175.1	LINC02188	ENSG00000260810.1	lnc-DGLUCY-1	ENSG00000226853.2	lnc-SCRN3–1	ENSG00000214546.3	lnc-PPP1R1B-1	ENSG00000228962.1	HCG23
ENSG00000234899.5	lnc-SLC39A11–10	ENSG00000230314.2	ELOVL2-AS1	ENSG00000213793.3	lnc-ZNF320–1	ENSG00000230033.1	lnc-NOM1–1	ENSG00000255248.2	MIR100HG
ENSG00000251161.2	lnc-VPS18–1	ENSG00000267715.1	lnc-REEP6–1	ENSG00000234703.1	lnc-CLIC6–3	ENSG00000272663.1	lnc-LHCGR-2	ENSG00000226237.1	GAS1RR
ENSG00000272430.1	lnc-SYT15–3	ENSG00000250101.1	lnc-FAM153C-5	ENSG00000232679.1	LINC01705	ENSG00000237883.1	DGUOK-AS1	ENSG00000231246.1	lnc-ST7L-2
ENSG00000257718.1	CPNE8-AS1	ENSG00000260219.1	lnc-MYLPF-1	ENSG00000259802.1	lnc-CMBL-1	ENSG00000236921.1	lnc-CDKN2B-1	ENSG00000248869.1	LINC02511
ENSG00000215190.4	lnc-RAB23–53	ENSG00000236703.1	MYB-AS1	ENSG00000266040.1	lnc-EPOP-2	ENSG00000255118.1	lnc-SCGB1D4–1	ENSG00000260947.1	lnc-PRSS3–2
ENSG00000272620.1	lnc-SORCS2–1	ENSG00000260136.1	lnc-SCNN1B-1	ENSG00000250081.1	lnc-ADAMTS6–1	ENSG00000262973.1	lnc-ABI3–6	ENSG00000237560.1	LINC01497
ENSG00000226419.2	SLC16A1-AS1	ENSG00000264589.1	MAPT-AS1	ENSG00000232940.1		ENSG00000254966.1	AC103974.1	ENSG00000263586.1	HID1-AS1
ENSG00000267287.1	lnc-NFATC1–1	ENSG00000249684.1	lnc-FAM153C-5	ENSG00000236591.1	lnc-ZC3H12D-2	ENSG00000259702.1	lnc-PGPEP1L-60	ENSG00000245812.2	LINC02202
ENSG00000253348.1	lnc-KCNMB1–3	ENSG00000254231.1	lnc-SLC7A13–1	ENSG00000263931.1	lnc-MARCH10–1	ENSG00000240497.2	lnc-TMEM212–1	ENSG00000238120.1	LINC01589
ENSG00000270170.1	NCBP2-AS2	ENSG00000268049.1	AC012313.2	ENSG00000228221.1	LINC00578	ENSG00000273328.1	lnc-KRBOX1–1	ENSG00000230587.1	LINC02580
ENSG00000233654.1	lnc-MFSD6–1	ENSG00000263105.1	lnc-SRL-1	ENSG00000244300.2	GATA2-AS1	ENSG00000273237.1	lnc-HNRNPA2B1–14	ENSG00000272316.1	lnc-RAB23–53
ENSG00000257271.1	KIRREL3-AS1	ENSG00000260954.1	lnc-PTX4–2	ENSG00000254854.1	lnc-RNF26–1	ENSG00000225243.1	lnc-MYOC-1	ENSG00000272143.1	FGF14-AS2
ENSG00000237976.1	lnc-PSMB4–1	ENSG00000228133.2	lnc-DPH1–1	ENSG00000231482.2	lnc-PXDN-2	ENSG00000226733.1	lnc-C6orf141–2	ENSG00000232079.2	LINC01697
ENSG00000253164.1	lnc-ATP6V1B2–3	ENSG00000228043.1	lnc-NXPH2–3	ENSG00000228950.1	lnc-RHOB-1	ENSG00000234072.1	lnc-SNX17–1	ENSG00000261625.1	lnc-MRGPRF-1
ENSG00000228109.1	MELTF-AS1	ENSG00000238122.1	lnc-NBPF4–1	ENSG00000261039.1	LINC02544	ENSG00000265282.1	lnc-CYB561–1	ENSG00000253864.1	CARMN
ENSG00000267480.1	lnc-MPPE1–10	ENSG00000260009.1	LINC02130	ENSG00000227857.2	lnc-TSPAN1–1	ENSG00000261712.1	lnc-KIAA1024–1	ENSG00000243243.1	lnc-TFEC-2
ENSG00000263893.2	lnc-SOX9–5	ENSG00000253959.1	LINC01863	ENSG00000233818.1	lnc-CHAF1B-2	ENSG00000259933.2		ENSG00000225731.1	lnc-WDR4–5
ENSG00000254148.3	lnc-SLC39A11–10	ENSG00000251169.2	LINC01843	ENSG00000224189.2	HAGLR	ENSG00000225833.1	lnc-BMX-1	ENSG00000229645.4	lnc-SYNE3–1
ENSG00000228639.2	lnc-SLC39A11–10	ENSG00000272933.1	lnc-ARL3–1	ENSG00000249917.2	LINC00536	ENSG00000258689.1	LINC01269	ENSG00000227467.3	LINC01537
ENSG00000245614.2	DDX11-AS1	ENSG00000271420.1	lnc-TCEA3–1	ENSG00000230479.1	lnc-CHAF1B-3	ENSG00000242540.2	lnc-SOX11–1	ENSG00000238018.1	lnc-RTN4–3
ENSG00000260420.1	LINC02182	ENSG00000248399.1	lnc-CFAP99–1	ENSG00000267493.2	CIRBP-AS1	ENSG00000261123.1	lnc-NTHL1–1	ENSG00000224318.1	CHL1-AS2
ENSG00000232803.1	SLCO4A1-AS1	ENSG00000268895.1	A1BG-AS1	ENSG00000223387.2	LINC02068	ENSG00000228288.2	PCAT6	ENSG00000272777.1	lnc-ADH5–1
ENSG00000229692.3	SOS1-IT1	ENSG00000262869.1	lnc-SMG6–4	ENSG00000264007.1	lnc-SSH2–1	ENSG00000245080.4	MIR3150BHG	ENSG00000231943.3	PGM5P4-AS1
ENSG00000232445.1	LNCPRESS1	ENSG00000263244.1	lnc-PMM2–6	ENSG00000267013.1	LINC01929	ENSG00000249199.1	lnc-MYO10–2	ENSG00000178947.8	SMIM10L2A
ENSG00000224930.2		ENSG00000224698.1	lnc-NBPF6–1	ENSG00000260766.1	lnc-PIGM-1	ENSG00000254248.1	lnc-TP53INP1–2	ENSG00000226833.1	lnc-TRMT61B-1
ENSG00000272825.1	lnc-FAM207A-2	ENSG00000267277.1	lnc-EPOR-2	ENSG00000267118.1	lnc-CCL4–3	ENSG00000253217.1	lnc-SPAG1–1	ENSG00000255850.1	RXYLT1-AS1
ENSG00000224717.1	lnc-CDK18–1	ENSG00000261742.1	LINC00922	ENSG00000264112.1	lnc-VEZF1–1	ENSG00000258616.1	LINC02303	ENSG00000180769.4	WDFY3-AS2
ENSG00000251136.4	lnc-NBN-1	ENSG00000234918.1	lnc-ZNF33A-13	ENSG00000260162.2	lnc-BANP-14	ENSG00000248161.1	lnc-SLC39A8–1	ENSG00000267107.2	PCAT19
ENSG00000254607.2	lnc-ST3GAL4–4	ENSG00000215256.3	DHRS4-AS1	ENSG00000263655.1	lnc-FBXO15–1	ENSG00000225077.2	LINC00337	ENSG00000259134.1	LINC00924
ENSG00000261189.1	lnc-CAGE1–1	ENSG00000272335.1	lnc-MRPS30–8	ENSG00000249942.1	lnc-BTC-5	ENSG00000226207.1	lnc-MMS22L-1	ENSG00000225039.1	LINC01058
ENSG00000248846.2	LINC02065	ENSG00000263280.1	lnc-PRSS22–1	ENSG00000238123.1	MID1IP1-AS1	ENSG00000230212.2	CBR3-AS1	ENSG00000228649.4	SNHG26
ENSG00000237857.2	lnc-FANCC-1	ENSG00000260793.2	lnc-TMUB2–1	ENSG00000196421.3		ENSG00000229272.1	lnc-EIF3A-2	ENSG00000229694.2	LINC00484
ENSG00000225096.1	lnc-PRIM2–7	ENSG00000224577.1	LINC01117	ENSG00000227543.3	SPAG5-AS1	ENSG00000260209.1	lnc-PUS3–1	ENSG00000225655.1	PGM5P3-AS1
ENSG00000185904.7	LINC00839	ENSG00000260526.1	lnc-TIFA-1	ENSG00000226876.2	lnc-KIF26B-2	ENSG00000253417.1	LINC02159	ENSG00000230747.1	lnc-SNRNP200–1
ENSG00000226101.1	LINC02097	ENSG00000233621.1	LINC01137	ENSG00000261327.3	lnc-CA5A-4	ENSG00000235939.1	lnc-C10orf71–1	ENSG00000253552.3	HOXA-AS2
ENSG00000203362.2	POLH-AS1	ENSG00000271916.1	lnc-CHDH-1	ENSG00000265908.1	lnc-PIPOX-5	ENSG00000272894.1	lnc-COL28A1–1	ENSG00000267082.1	lnc-ANGPTL8–1
ENSG00000236663.1	FRGCA	ENSG00000231868.1	lnc-TNFRSF25–1	ENSG00000254239.1	lnc-SLC25A48–1	ENSG00000260231.1	KDM7A-DT	ENSG00000226031.1	FGF13-AS1
ENSG00000241155.1	ARHGAP31-AS1	ENSG00000272913.1	lnc-TRIM43B-1	ENSG00000153363.8	LINC00467	ENSG00000233342.1	lnc-MAP3K4–2	ENSG00000227591.1	lnc-LAMB3–1
ENSG00000272048.1	lnc-DNMT3A-1	ENSG00000273472.1	lnc-ELMOD2–4	ENSG00000258457.1	lnc-PSMB11–1	ENSG00000258829.1	lnc-DIO2–5	ENSG00000231999.2	LRRC8C-DT
ENSG00000239445.1	ST3GAL6-AS1	ENSG00000223813.2	lnc-CPVL-4	ENSG00000264083.1	lnc-PSMD11–1			ENSG00000261472.1	lnc-WWOX-1
ENSG00000261521.1	lnc-DSC2–1	ENSG00000255367.1	lnc-ART5–1	ENSG00000242136.1	lnc-ASAP2–4			ENSG00000186594.8	MIR22HG
ENSG00000237594.2	lnc-SOD1–3	ENSG00000270177.1	lnc-TCF7–4	ENSG00000268686.1	lnc-TEAD2–1			ENSG00000255471.1	lnc-FZD4–1
ENSG00000234753.1	FOXP4-AS1	ENSG00000267009.2	lnc-ARSG-3					ENSG00000224468.3	LAMC1-AS1

### LINC01614 elevated expression is associates with enhanced BC tumorigenenic molecular profile

**Fig. 5:**
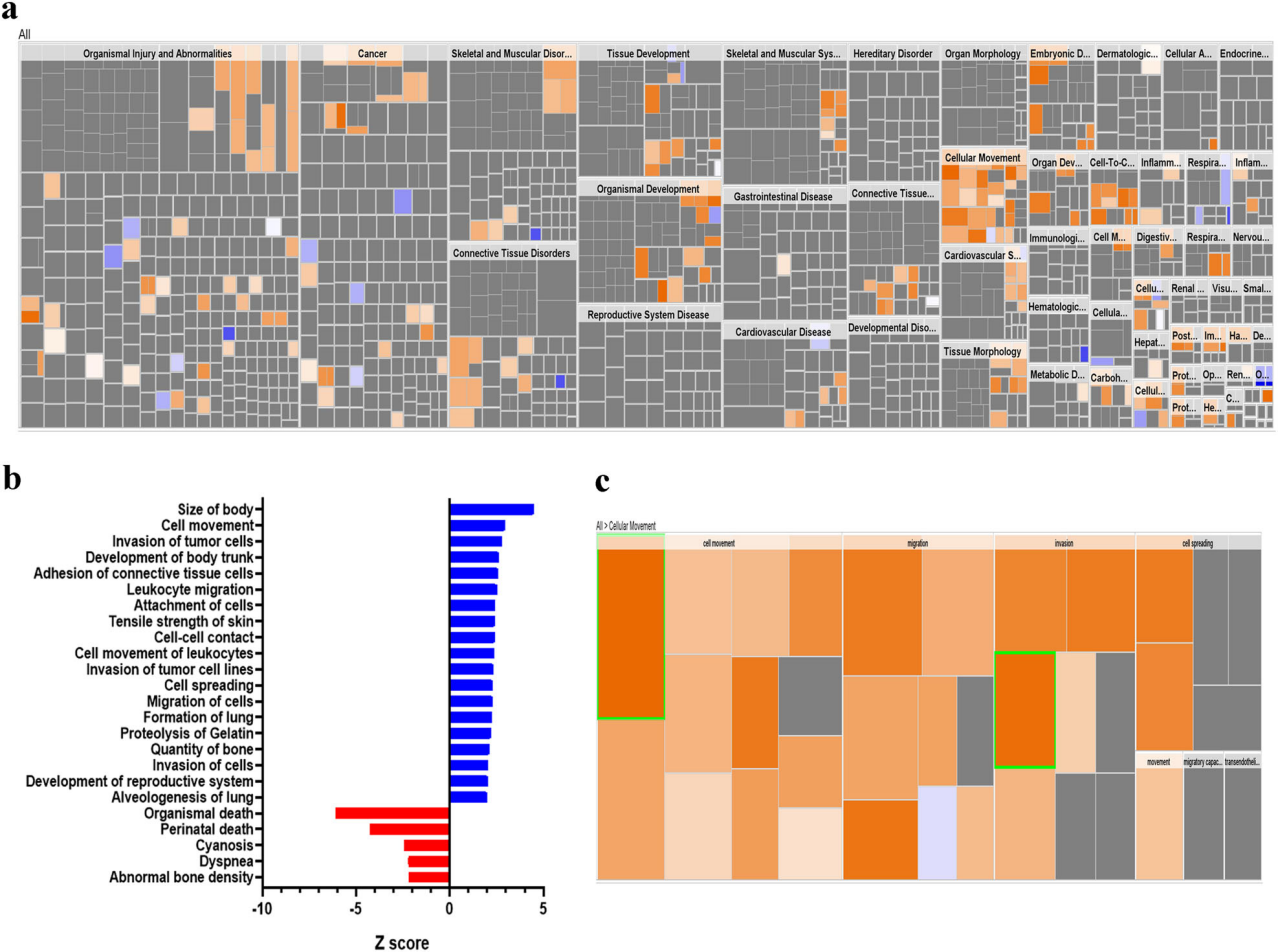
Enrichment in tumor cell migration and invasion functional categories in LINC01614^high^ BC patients. **a** Disease and function heat map depicting enrichment in the indicated functional and disease categories in the upregulated transcripts in LINC01614^high^ vs LINC01614^low^ BC patients based on IPA analysis. **b** Bar-graph depicting the most significantly affected factional categories in LINC01614^high^ vs LINC01614^low^ BC patients. **c** Heat map-illustrating enrichment in cellular movement functional category

To gain more insight into plausible role for LINC01614 in BC pathology, the 837 BC patients were divided into LINC01614^high^ and LINC01614^low^ according to the median LINC01614 expression. The upregulated genes in the LINC01614^high^ group were subsequently subjected to Ingenuity Pathway Analysis (IPA) and downstream effect analysis (DEA). Affected functional categories are illustrated as heat tree map, which clusters functionally associated categories together, therefore depicting a high-level outlook of enriched functional categories. Data presented in Figs. 5a, b revealed a number of enriched functional categories including those involved in cell movement and invasion, while functional categories associated with cell death were under presented. Illustration of the cellular movement functional category is shown in Fig. 5c.

### Mechanistic network analysis predicts activation of TGFβ1 and ECM pathways in LINC01614^high^ BC tissue

**Fig. 6:**
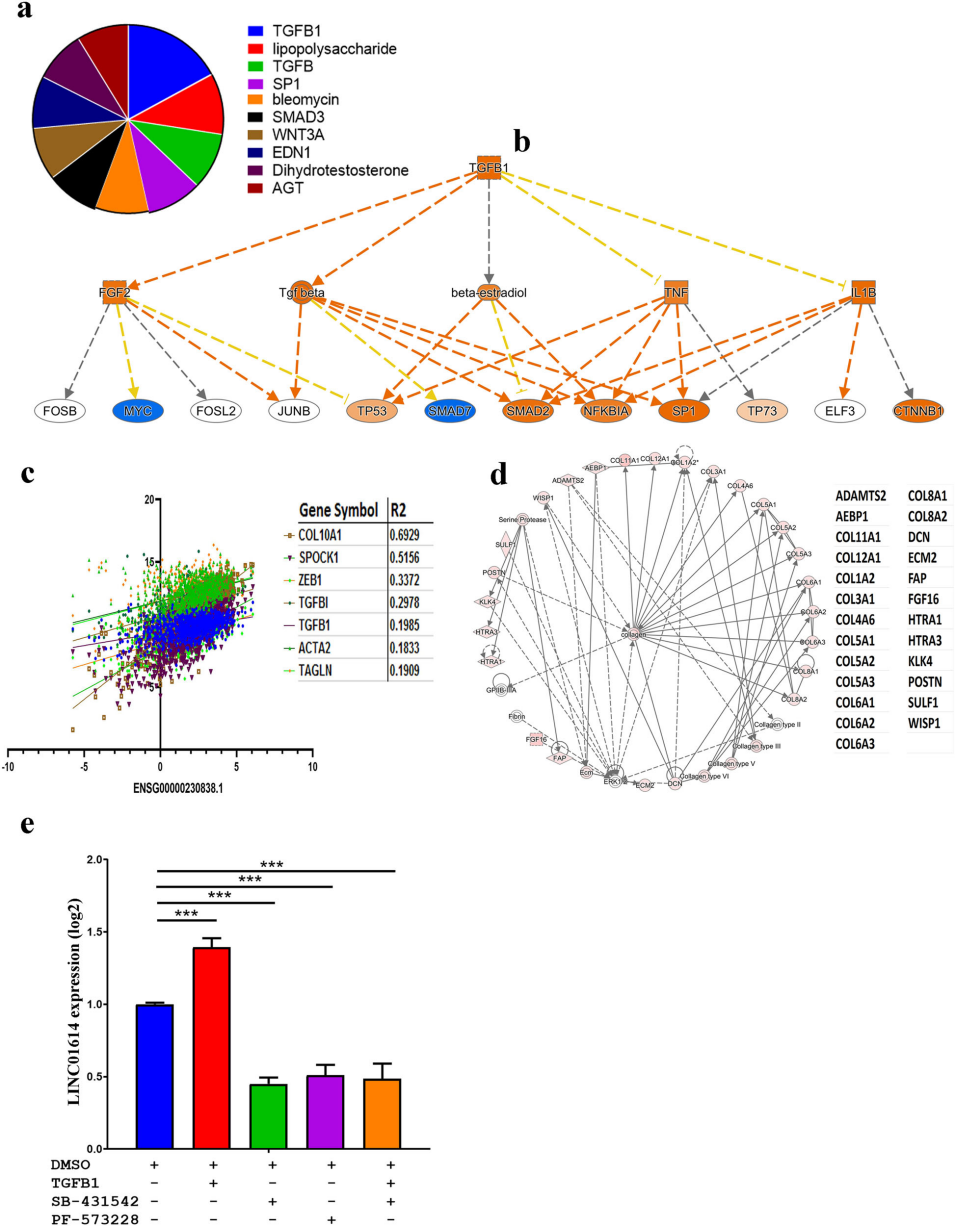
Mechanistic network analysis predicts predominant activation of the TGFB pathway in LINC01614^high^ BC tumors. **a** Pie chart illustrating the top activated mechanistic networks in LINC01614^high^ BC tumors based on IPA analyses. Segment size corresponds to the activation *Z* score**. b** Illustration of the TGFβ1 signaling network. **c** Correlation between the expression of LINC01614 and the expression several members of the TGFβ family in BC tumors. **d** Extra cellular matrix functional enrichment in LINC01614^high^ BC tumors. Color intensity indicates their activation state. Effect of recombinant TGFβ (10 ng/ml), SB-431542 (ββ inhibitor, 10 µM), and PF-573228 (FAK inhibitor, 5 µM) on LINC01614 expression measured by qRT-PCR. Data are presented as mean ± S.E. from two independent experiments, *n* = 6

Upstream regulator analysis (URA) on the upregulated genes in LINC01614^high^ revealed significant enrichment for a number of networks including TGFβ1, lipopolysaccharide, TGFβ, SP1, bleomycin, SMAD3, WNT3A, EDN1, dihydrotestosterone, and AGT (Fig. 6a). Highest enrichment was for the TGFβ1 network (*Z* score = 5.6; Fig. 6a). Mechanistic network analysis predicted TGFβ1 to directly activate the SMAD2, NFKB1A and SP1 through TGFβ (direct activation) and TNF (inconsistent sate), and to inhibit MYC through FGF2 (direct activation) and inhibit SMAD7 through TGFβ with higher confidence level (Fig. 6b). Concordantly, LINC01614 expression demonstrated significant positive correlation with various members of the TGFβ signaling pathways (COL10A1 (*R*^2^ = 0.7), SPOCK1 (*R*^2^ = 0.5156), ZEB1 (*R*^2^ = 0.3372), TGFBI (*R*^2^ = 0.2978), TGFB1 (*R*^2^ = 0.1985), ACTA2 (*R*^2^ = 0.1833), and TAGLN (*R*^2^ = 0.1909)) in the TCGA BC cohort (Fig. 6c). Moreover, we observed several collagen family members to be upregulated in LINC01614^high^ BC, suggesting enhanced extracellular matrix (ECM) formation. Illustration of the ECM network in LINC01614^high^ BC mapped by IPA is shown in Fig. 6d. The color shade intensity of the node correlates with the expression level of the indicated genes. Therefore, our molecular and network analyses revealed strong correlation between LINC01614 expression, TGFβ and ECM signaling. Mechanistically, recombinant TGFβ1 induced LINC01614 expression, while pharmacological inhibition of TGFβ signaling (using SB-431542) and FAK (using PF-573228) inhibited LINC01614 expression in BC cells (Fig. 6e), thus implication TGFβ and FAK signaling in regulating LINC01614 expression in BC cells.

## Discussion

In recent years, lncRNAs have emerged as key players in regulating cellular functions, differentiation and disease progression, including cancer, through epigenetics, chromatin remodeling, transcriptional and post-transcriptional regulation^5,14^.

While the number of annotated lncRNAs in the human genome has increase dramatically, functional characterization of lncRNAs and their utilization as disease biomarkers is begging to unfold. In current study, we analyzed the lncRNA transcriptome from the TCGA breast cancer dataset and performed thorough survival and bioinformatics analyses which revealed eighteen upregulated and forty-six downregulated lncRNAs in BC compared to normal breast tissue. Additionally, our data identified different lncRNA signatures associated with various BC molecular subtypes (HER2^+^HR^+^, HER2^+^HR^−^, HER2^−^HR^+^, and TNBC) as well as those specific to normal breast tissue. Interestingly, our data revealed a distinct lncRNA cluster for the TNBC tumors, while such segregation was less evident among the other molecular subtypes (HER2^+^HR^+^, HER2^+^HR^−^, HER2^−^HR^+^). This could be attributed to the tissue of origin for various BC molecular subtypes, where TNBC originates from ductal cells, while the HR+ and HER2+ originate from luminal cells^15^.

Our analyses identified eleven lncRNAs (LINC01614, LINC01235, lnc-LRR1–1, lnc-ODF3B-2, AC015712.5, lnc-LAMB3–1, lnc-SPP2–3, lnc-MAP9–2, MIR205HG, lnc-MAP2K6–5 and FGF14-AS2) whose expression correlated with patient outcome. Among the identified lncRNAs, LINC01614 and LINC01235 correlated with worse DFS, while LINC01235 correlated with worse OS. Interestingly, LINC01235 was downregulated in in BC compared to normal tissue, while at the same time it predicted worse DFS and OS. It is plausible that due to the large heterogeneity of BC cases included the TCGA BC cohort, the expression pattern for LINC01235 did not correlate with survival data. Additionally, a previous study reported LINC01235 (also called FLJ41200; ENSG00000270547.1) as cancer-related genes that mapped telomeric and centromeric to CD274 (PDL-1) at 9p23 in small-cell lung carcinoma^16^, suggesting possible link between LINC01235 and immune regulation in cancer.

Interestingly, our data revealed over-expression of LINC01614 in BC compared to normal tissue and its elevated expression correlated with worse DFS. More in-depth analysis revealed LINC01614 to be highly expressed in ER^+^ (log2 exp = 2.1), in PR^+^ (log2 exp = 2.2) and HER2^+^ (log2 exp = 2.623), while TNBC exhibited lowest expression (log2 exp = 1.1). Those data were further validated in a second cohort where highest expression was observed in the luminal B/HER2^+^ molecular subtype while lowest expression was observed in the HER2-TNBC molecular subtype. The expression of LINC01614 did not correlate with BC disease stage, suggesting alteration in LINC01614 expression as an early feature during BC development and progression. Concordant with our data, LINC01614 expression has been linked to lung adenocarcinoma^17^ and the LINC01614-contaitng signature predicted OS and DFS in patients with esophageal squamous cell carcinoma^18^. Recently, LINC01614 has also been reported as one of the lncRNA associated survival of ER + BC patients^19^.

To gain more insight into plausible molecular mechanisms of LINC01614 expression and function, we dicatomized the TCGA BC cohort into LINC01614^high^ and LINC01614^low^ and subsequently retrieved and identified mRNA transcripts upregulated in the LINC01614^high^ group, which revealed 187 upregulated transcripts. Interestingly, IPA analysis on the upregulated gene list suggested strong correlation between LINC01614 expression and enriched functional categories associated with tumor cell movement and invasion. Nonetheless, LINC01614^high^ expression was most significantly associated with TGFβ signaling, suggesting possible induction of LINC01614 by TGFβ signaling. Additionally, LINC01614^high^ tumors exhibited high expression of several collagens, suggesting possible association between LINC01614 expression and enhanced ECM formation. It is noteworthy that ECM itself could be regulated by TGFB signaling^20^. Mechanistic investigation validated induction of LINC01614 by TGFβ, while it’s expression was inhibited by small molecule inhibitor of TGFβ and FAK, suggesting its regulation by TGFβ and FAK signaling.

Our data also revealed elevated expression of LINC01614 in HER2^+^ BC tumors. Interestingly, we observed significant correlation between LINC01614 expression and HER2 mutation status in BC (supplementary figure 1). HER2^+^ (erbB2) represent 25 to 30 % of breast cancer patients and is elevated expression has been associated with more aggressive BC phenotype and shorter DFS and OS^21,22^. Additionally, activation of HER2 has been linked to Epithelial-to-mesenchymal transition (EMT), hence endowing cancer cells with a more aggressive and invasive phenotype^23,24^. Interestingly, HER2 and TGFβ signaling cooperated in the induction of cellular processes associated with tumorigenic development in immortalized mammary epithelial cell line^25^. Additionally, overexpression of HER2 activated the TGFβ/SMAD signaling pathway and induced SNAIL, SLUG and ZEB-1 expression and subsequent acquisition of mesenchymal phenotype^24^. These published reports are consistent with our current data linking LINC01614 to TGFβ signaling and HER2 + molecular subtype.

## Conclusions

Our data revealed the lncRNA transcriptional landscape in breast cancer and identified the lcnRNA signatures associated with each molecular subtype. Specifically, our data provide novel insight implicating LINC01614 as unfavorable prognostic marker in BC, and its association with the HR^+^/HER2^+^ BC molecular subtype and its regulation by TGFβ and FAK signaling.

## Supplementary information

Supplementary figure 1
