# Supplementary material for: Long non-coding RNA (lncRNA) transcriptional landscape in breast cancer identifies LINC01614 as non-favorable prognostic biomarker regulated by TGFβ and focal adhesion kinase (FAK) signaling
Source: Cell Death Discov. 2019 Jun 24;5:109. doi: 10.1038/s41420-019-0190-6 (PMC6591245; doi:10.1038/s41420-019-0190-6)
Supplement: Supplementary file 1 — Supplementary figure 1 [file 41420_2019_190_MOESM1_ESM.docx]

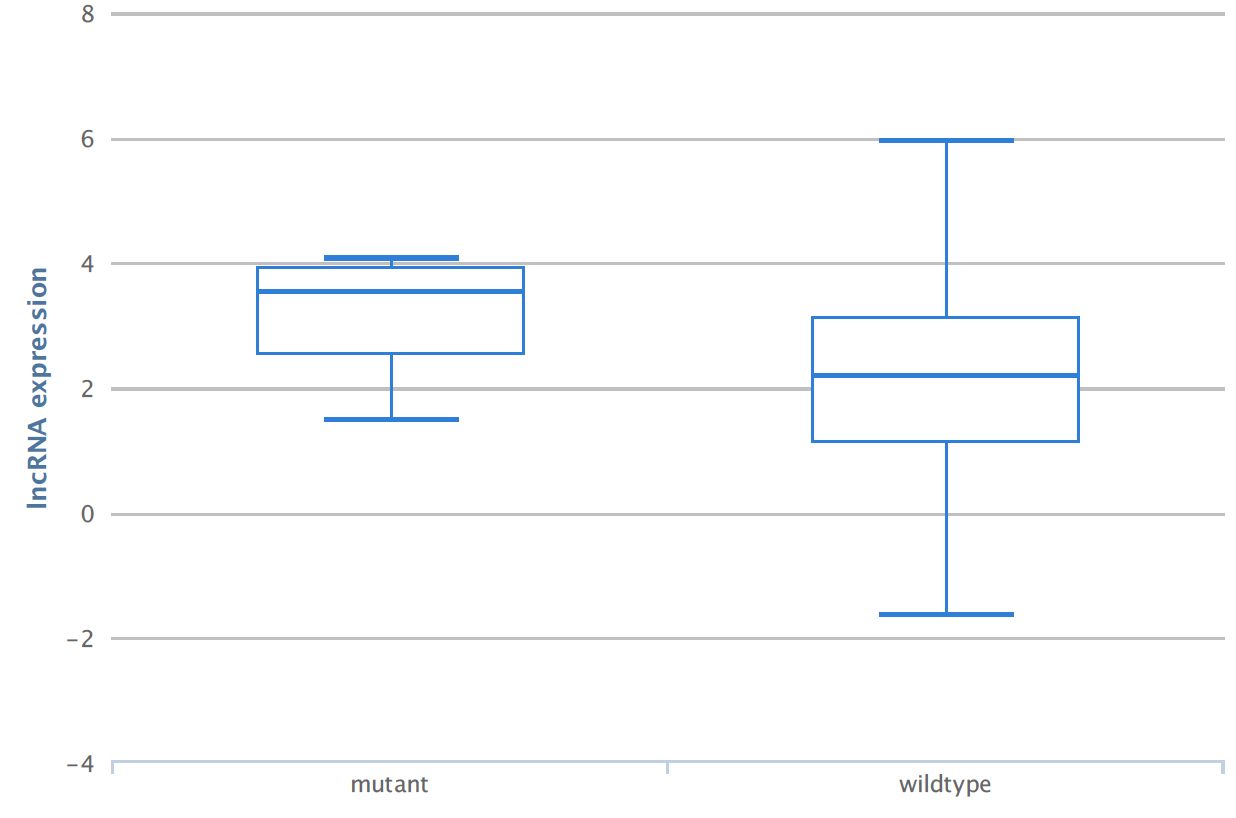


**Supplementary figure 1.** Expression of [LINC01614](https://mail.qf.org.qa/owa/redir.aspx?C=ffQayIKnck1l-1s71OyNcVHd3YBpKyF9y1uU9J6S9McjI-r68I_WCA..&URL=http%3a%2f%2fasia.ensembl.org%2fhomo_sapiens%2fGene%2fSummary%3fg%3dENSG00000230838%26db%3dcore) in breast cancer with ERBB2 mutation compared to those with wild type ERBB2 from the TCGA dataset. Mutant mean (3.0), wild type mean (1.99), p = 0.005.
